# Prevalence of musculoskeletal symptoms in obese patients candidates for bariatric surgery and its impact on health related quality of life

**DOI:** 10.1590/2359-3997000000237

**Published:** 2017-01-27

**Authors:** Gabriela Calenzani, Fabiana Ferreira dos Santos, Veronica Lourenço Wittmer, Grace Kelly Filgueiras Freitas, Flávia Marini Paro

**Affiliations:** 1 Departamento de Educação Integrada em Saúde Universidade Federal do Espírito Santo Vitória ES Brasil Departamento de Educação Integrada em Saúde, Universidade Federal do Espírito Santo (UFES), Vitória, ES, Brasil

**Keywords:** Obesity, quality of life, musculoskeletal pain, morbid obesity, physical therapy, epidemiology

## Abstract

**Objective:**

This study was designed to identify the major musculoskeletal symptoms of individuals with obesity, to assess their health-related quality of life, and to evaluate the correlation between the musculoskeletal symptoms and the individuals’ health-related quality of life.

**Materials and methods:**

Cross-sectional study.

**Instruments used:**

“Nordic Musculoskeletal Questionnaire” and “The Medical Outcomes Study 36-Item Short-Form Health Survey (SF-36)”.

**Results:**

In total, 41 subjects were evaluated, of which 90.15% were female. The mean age of the subjects was 40.78 ± 9.85 years and their mean body-mass index was 46.87 ± 8.08. All subjects reported musculoskeletal pain in at least one anatomical region and 80.49% had pain in three or more regions. The activity limitations due to pain were reported by 75.61% of them. The most affected regions by pain were the ankles and/or feet, lower back, knees and wrists/hands/fingers. The most associated regions with activity limitations due to pain were the ankles and/or feet, knees and lower back. The presence of pain showed a negative correlation with the domains physical functioning (PF), role-physical (RP) and body pain (BP). The activity limitations showed a negative correlation with the domains PF, BP, social functioning (SF) and role-emotional (RE).

**Conclusion:**

Our data showed a high prevalence of musculoskeletal pain and limitation in activities due to pain in obese subjects. The musculoskeletal symptoms had negative correlations with physical and mental components of the health-related quality of life, highlighting the importance of ensuring that patients with obesity have access to interdisciplinary care, for the prevention and rehabilitation of musculoskeletal disorders.

## INTRODUCTION

The incidence of obesity has increased in recent decades, and obesity has become a major global health challenge (1). In Brazil, approximately 50% of men and women over 20 years are overweight, and the prevalence of obesity is 12.5% for men and 16.9% for women (2). Increased body-mass index (BMI) is an important risk factor for several causes of mortality, including cardiovascular disease, many types of cancer, hepatic and kidney diseases, and others (3).

A higher prevalence of musculoskeletal symptoms affecting mainly the lower limbs has been reported in obese individuals (4,5). Some of these symptoms manifest as joint discomfort caused mainly by excess weight, which can overload the musculoskeletal structures responsible for stabilizing and moving the body (6). Obesity has been associated with significant impairment in mobility (7) and several musculoskeletal disorders, which can lead to disability and poor quality of life (5,6) and result in high direct and indirect healthcare economic costs (8,9).

Some studies have reported that obesity has a negative impact on the patients’ health-related quality of life (10,11) but only a few studies have assessed the impact of musculoskeletal symptoms on the quality of life of obese individuals (12).

The purpose of this study was to identify the major musculoskeletal symptoms of individuals with obesity grades II, III and IV, to assess their health-related quality of life, and to evaluate the correlation between the musculoskeletal symptoms and the patients’ quality of life. This information will be useful for the development of preventive and therapeutic measures to improve the musculoskeletal symptoms and quality of life of obese patients.

## MATERIALS AND METHODS

This was a cross-sectional study and was approved by the Ethics Committee of the Universidade Federal do Espírito Santo (approval number 293/10). The data were collected from April to December 2011. The study population consisted of all patients aged over 18 years who underwent preoperative consultations for bariatric surgery in a Brazilian University Hospital during the study period (n = 41).

During the physical therapy consultation of the patients were collected general data, such as age, sex, height, weight, marital status, education, occupation and lifestyle habits. Two questionnaires were administered by the researchers, who were properly trained so that the assessments were standardized. The first questionnaire was the validated Brazilian version of the Nordic Musculoskeletal Questionnaire (NMQ) (13,14), which consists of multiple-choice or binary responses to questions regarding the occurrence of symptoms in anatomical regions. This questionnaire covers the 12 months and the 7 days preceding the assessment, as well as the withdrawal from routine activities due to musculoskeletal symptoms in the 12 months preceding the assessment. The QNSO has been used in other studies of obese individuals (15,16).

Immediately after, we used “The Medical Outcomes Study 36-Item Short-Form Health Survey” (SF-36), which was validated and translated into Portuguese (17,18), for the evaluation of the patients’ health-related quality of life. This a multidimensional questionnaire consisting of 36 items, which are grouped into eight scales or domains, four of which include components related to physical health, namely, physical functioning (PF), role-physical (RP), bodily pain (BP), and general health (GH), and four of which include components related to mental health, namely, vitality (VT), social functioning (SF), role-emotional (RE) and mental health (MH). The answers to the questions in each domain are assigned numerical score, which is coded and classified on a scale from 0 to 100. The higher the score, the better the health-related quality of life in that domain (18).

Data were analyzed using the Statistical Package for the Social Sciences (SPSS) 18.0, IBM. To measure the degree of association between two metric variables, we used the correlation coefficient. The Shapiro-Wilk test was used to determine whether the data distribution was normal. For variables where the normal hypothesis was not rejected, the t-test was used to analyze the means (parametric analysis). For the domains where the assumption of normality was rejected, we applied the nonparametric Mann-Whitney test. To assess correlations, the Spearman correlation coefficient was used. The data were expressed with standard deviations (SD) and differences were considered significant if p < 0.05.

## RESULTS

We evaluated 41 obese subjects (grades II to IV), of whom 37 (90.2%) were female and four (9.85%) were male, candidates to bariatric surgery. The average age of the patients was 40.78 ± 9.85 years and ranged from 25 to 65 years. The patients’ mean BMI was 46.87 ± 8.08 kg/m^2^ and ranged from 35.73 to 65.02 kg/m^2^. All subjects were followed up by the health interdisciplinary team of the Bariatric Surgery Program in the hospital.

The most frequent comorbidities were systemic arterial hypertension (SAH), which was observed in 31 subjects (70.7%), and diabetes mellitus (DM), which was observed in 16 subjects (39%). All individuals with DM also had hypertension. In terms of physical activity, 68.3% reported that they did not participate in any physical activity. Of the 28.3% who performed some activity, 26.8% participated in walking, with frequency ranging from two to five times a week, and 2.4% participate in aquatic exercises, with frequency ranging two to five times a week.

### Prevalence of musculoskeletal symptoms

In the 12 months preceding the questionnaire, 100% of subjects reported pain in at least one anatomical region and 80.49% had pain in 3 or more anatomical regions. In the previous 7 days, 92.68% reported pain in at least one anatomical region and 60.98% in 3 or more regions. The limitation of activities due to pain in the previous 12 months was reported by 75.61% of patients. Table 1 shows the detailed distribution of patients by number of anatomic regions affected.

**Table 1:** Distribution of subjects by number of anatomical regions affected by musculoskeletal symptoms according to the NMQ

Affected anatomical regions (n)	Pain (last 12 months)		Pain (last 7 days)		Limitation of activities (last 12 months)	
		
	n	%	n	%	n	%
None	0	0	3	7.32	10	24.39
1	3	7.32	5	12.20	6	14.63
2	5	12.20	8	19.51	4	9.76
3	5	12.20	6	14.63	8	19.51
4	7	17.07	4	9.76	5	12.20
5	5	12.20	4	9.76	2	4.88
6	5	12.20	4	9.76	2	4.88
7	4	9.76	4	9.76	3	7.32
8	6	14.63	3	7.32	0	0
9	1	2.44	0	0	1	2.44

NMQ: Nordic Musculoskeletal Questionnaire.

The anatomical regions most affected by pain in the previous 12 months were the ankles/feet (87.80%), lumbar region (68.29%) and knees (68.29%). In the preceding 7 days, 70.73% had pain in the ankles/feet, 53.66% in the lumbar region, 46.34% in knees and 46.34% in the knees, wrists/hands/fingers. The regions that were most frequently associated with activity limitations due to pain were the ankles/feet (46.34%), knees (43.90%) and lower back (41.46%). The prevalence of pain by anatomic region is presented in detail in Table 2.

**Table 2:** Prevalence of musculoskeletal symptoms by anatomic region according to the NMQ

Anatomical regions	Pain (last 12 months)		Pain (last 7 days)		Limitation of activities (last 12 months)	
		
	n	%	n	%	n	%
Neck	16	39.02	10	24.39	4	9.76
Shoulders	19	46.34	15	36.59	10	24.39
Elbows	6	14.63	4	9.76	3	7.32
Forearm	7	17.07	6	14.63	7	17.07
Wrists/hands/fingers	25	60.98	19	46.34	11	26.83
Dorsal region	22	53.66	18	43.90	13	31.71
Lower back	28	68.29	22	53.66	17	41.46
Hips and/or thighs	11	26.83	9	21.95	8	19.51
Knees	26	63.41	19	46.34	18	43.90
Ankles/feet	36	87.80	29	70.73	19	46.34

NMQ: Nordic Musculoskeletal Questionnaire.

There was no statistically significant correlation between BMI and musculoskeletal symptoms. There was also no significant correlation between age and musculoskeletal symptoms.

### Health-related quality of life

Table 3 contains the descriptive statistics for the SF-36. The domain SF showed the highest score (61.95) and RP showed the lowest (42.68).

**Table 3:** Scores for the SF-36 domains

Domains	Mean	SD	CI	
Physical functioning (PF)	46.83	28.01	38.25	55.40
Role-physical (RP)	42.68	41.92	29.85	55.52
Bodily pain (BP)	46.93	19.59	40.93	52.92
General health (GH)	61.95	22.83	54.96	68.94
Vitality (VT)	44.15	25.07	36.47	51.82
Social functioning (SF)	65.85	32.24	55.99	75.72
Role-emotional (RE)	56.10	39.75	43.93	68.26
Mental health (MH)	55.80	27.76	47.31	64.30

SD: standard deviation; CI: confidence interval.

Table 4 shows the correlation results between SF-36 results, BMI and age. PF was the only domain that showed a statistically significant correlation with age (p = 0.007). Specifically, PF correlated negatively with age, meaning that the older the patient, the poorer the quality of life in this domain. None of the domains showed a statistically significant correlation with BMI.

**Table 4:** Correlations of the SF-36 domains with BMI and age

Domains (SF-36)	Age		BMI	
	
	Correlation coefficient	p-value	Correlation coefficient	p-value
Physical functioning (PF)	-0.418^1^	0.007	-0.188	0.239
Role-physical (RP)	-0.218	0.171	-0.092	0.568
Bodily pain (BP)	-0.229	0.15	-0.001	0.995
General health (GH)	-0.123	0.445	-0.002	0.991
Vitality (VT)	0.011	0.946	-0.117	0.467
Social functioning (SF)	-0.036	0.824	0.024	0.881
Role-emotional (RE)	-0.225	0.158	0.086	0.594
Mental health (MH)	0.057	0.725	-0.064	0.693

^1^ Statistically significant.

### Association between musculoskeletal symptoms and quality of life

The presence of pain in the previous 12 months showed a statistically significant correlation with the domains PF and BP. These correlations were negative, indicating that the greater the pain, the worse the quality of life in these domains. The presence of pain in the previous 7 days showed a significant negative correlation with the domains PF, RP and BP (Table 5). Table 5 shows that activity limitations showed a statistically significant correlation with poorer quality of life in the domains RP, BP, SF and RE.

**Table 5:** Correlations between the SF-36 domains and the presence of musculoskeletal symptoms evaluated by the NMQ

Domains	Pain (last 12 months)		Pain (last 7 days)		Limitations in activities (last 12 months)	
		
	Correlation coefficient	p-value	Correlation coefficient	p-value	Correlation coefficient	p-value
Physical functioning (PF)	-0.323^1^	0.039	-0.4951	0.001	-0.28	0.077
Role-physical (RP)	-0.21	0.187	-0.3321	0.034	-0.418^1^	0.007
Bodily pain (BP)	-0.312^1^	0.047	-0.4751	0.002	-0.455^1^	0.003
General health (GH)	-0.065	0.685	-0.124	0.441	-0.047	0.771
Vitality (VT)	-0.139	0.386	-0.177	0.269	-0.162	0.312
Social functioning (SF)	-0.166	0.299	-0.201	0.208	-0.319^1^	0.042
Role-emotional (RE)	0.012	0.94	-0.023	0.886	-0.364^1^	0.019
Mental health (MH)	-0.064	0.69	-0.164	0.307	-0.12	0.453

NMQ: Nordic Musculoskeletal Questionnaire; ^1^ Statistically significant.

## DISCUSSION

The results showed that all individuals with obesity of grades II to IV and who were candidates for bariatric surgery had musculoskeletal symptoms, and all subjects reported pain in at least one anatomical region in the 12 months preceding the assessment. The prevalence of pain in the 7 days preceding the assessment was also high (92.68%). A similar prevalence was found in another study, in which 100% of the obese individuals evaluated during preoperative bariatric surgery had musculoskeletal symptoms in at least one region of the body (16).

The current study demonstrated that the anatomical areas most often affected by pain in the 12 months and 7 days preceding the assessment were the ankles and/or feet, lower back, knees and wrists/hands/fingers. A similar prevalence of musculoskeletal symptoms in the ankles/feet and knee was shown in a study conducted in obese patients before and after bariatric surgery. Six months after surgery, the subjects experienced a marked reduction in both frequency and intensity of musculoskeletal pain, which strengthens the association between the symptoms and obesity. However, the study used the modified NMQ, considering only the questions about the presence of pain in the lower limbs in the previous 7 days (19). In addition, their results differed from our with regard to the high prevalence of hip pain (54.5%) compared with that observed in our population (21.95%). This is in accordance with a study that did not find any association between BMI and the occurrence of hip osteoarthritis (20).

Larsson’s results also showed that obese women had more musculoskeletal pain in the lower backs, knees and feet (15). The prevalence of musculoskeletal symptoms in the knees of obese people has been well-established in the literature (5,21). It has been demonstrated that weight gain is associated with adverse effects on the knees, such as pain, stiffness and functional changes (22). Additionally, it has reported that BMI > 30 kg/m^2^ is an important risk factor for the incidence of knee osteoarthritis (20).

In our study, back pain was the second most prevalent complaint, with a reported incidence of 68.29% in the past 12 months and 53.66% in the previous 7 days. A meta-analysis published in 2010 showed that obesity increases the risk of lower back pain and is also strongly associated with increased need for health care for the treatment of acute and chronic lower back pain (23). The association between obesity and lower back pain has also been shown in other studies (5,21).

Although only a few publications have related obesity to the prevalence of pain in wrist/hand/fingers, a cohort study conducted with 1,675 individuals showed that obesity was a significant independent predictor for the incidence of hand osteoarthritis (22). While the presence of osteoarthritis was not assessed in our study, this finding could be related to the high prevalence of pain in this region found in our results. In addition, obesity is considered an important risk factor for carpal tunnel syndrome (24).

A high prevalence of activity limitation due to pain was observed in the current study, and the anatomical regions most frequently associated with limitations were the ankles/feet (46.34%), knees (43.90%) and lower back (41.46%). These findings corroborate Larsson’s conclusion that high BMI value, age and lower body pain could predict functional limitations in obese women (25). Furthermore, it has been demonstrated that increased BMI is strongly associated with foot pain and disability, and this may impair both biomechanical and metabolic mechanisms (26).

With regard to quality of life, a study that also used the SF-36 showed a relationship between quality of life and obesity and identified an association between excess weight and low values in the domains PF, RP and BP (27). We must emphasize that these three domains and domain VT in the current study showed the lowest scores in the SF-36, which means that the lowest values were observed in the domains related to physical health components of health-related quality of life. Despite the fact that low values were also observed for mental health components, they were higher than the values for the physical health components. Cameron and cols*.* showed that obesity was related with impairment in both physical and mental components of health-related quality of life in Australian adults followed over 5 years (28). One factor that could explain the higher values for mental health components found in the current study is the fact that all patients in this study had been receiving psychological care during the months preceding the surgery because this service is part of the bariatric surgery program at our institution. In addition to this, the patients were evaluated a few weeks prior to bariatric surgery after months or years of waiting and preparation, because the study was conducted in a public hospital where there is a queue for bariatric surgery. As such, the expectation of the positive outcome of surgery in the next weeks possibly influenced the mental state of the patients. However, these patients did not receive any health professional care related to the prevention or treatment of musculoskeletal symptoms in the period before surgery.

A study conducted with 5,633 people in Sweden showed that the impact of obesity differs by age with relation to physical and mental health domains (25). In our results, there was a statistically significant negative correlation only between age and PF, which was possibly due the small sample size. This suggests that the older obese people had a worse quality of life in this domain than younger obese people.

We found no statistically significant correlation between the SF-36 and BMI in our study. However, in the similar study mentioned earlier (27), there were statistically significant negative correlations between BMI and each one of the domains PF, RP and BP. An important difference between this study and ours is that they compared groups with different features. Specifically, group I comprised overweight patients with obesity grades I and II, and group II comprised patients with grade III obesity in the earlier study. In contrast, our sample was composed only of severely obese (grade II), morbidly obese (grade III) or super obese (grade IV) patients, all of whom had indications for bariatric surgery. This means that even the obese grade II individuals presented comorbidities or risk factors that indicated them for surgery. It is also possible that in our study, the correlation between BMI and quality of life did not show significance because our population is less heterogeneous. Another study showed that the degree of obesity had no impact on knee osteoarthritis symptoms of pain, stiffness and functional difficulty; and, in two functional capacity tests, both obese women and women with morbid obesity (29).

Sach and cols. reported that the higher the BMI, the worse the HRQL of individuals compared to those with normal BMI (30). Cameron and cols. demonstrated that a bidirectional relationship was observed between the decline of quality of life and increase in weight. Accordingly, reduced quality of life increases the likelihood of increasing weight, and increased weight leads to reduced quality of life (28).

Some studies in the literature have reported that musculoskeletal pain is a predictor of poor health-related quality of life in obese subjects (5,12,31), but only a few of these studies have correlated the presence of pain with different health-related quality of life domains in obese individuals, and they haven’t correlated the limitation of activities due to pain with the different domains of health-related quality of life.

The present study showed a significant negative correlation between the presence of pain and the reduction in quality of life related to health in the domains PF, RP, BP. In other words, the musculoskeletal pain in these obese patients had a negative impact on their quality of life in these domains, suggesting that in our study, the physical health component of the individuals’ health-related quality of life was more affected by pain than the mental health component. In addition to this, the results also indicated that the limitation of activities by pain had a statistically significant negative correlation with the domains RP, BP, SF and RE, meaning that limitation of activities due to pain negatively affected both the physical and the mental components of health-related quality of life. Such knowledge can provide evidence to support the adoption of primary, secondary, tertiary health care prevention, and interdisciplinary rehabilitation, to ensure more effective interventions for these patients.

The major limitations of this study were the small sample size and the fact that the study was conducted in a single institution. Due to both of these features, generalized conclusions cannot be drawn from this study. Another limitation is that the causes of the musculoskeletal symptoms were not investigated by clinical and radiological evaluation but only by questionnaire. Finally, we did not evaluate other comorbidities that could interfere with the patients’ health-related quality of life.

In conclusion, individuals with obesity grades II, III and IV who were candidates for bariatric surgery showed high prevalence of musculoskeletal symptoms and of activity limitation by pain. They also had low scores in all domains of health-related quality of life. Furthermore, the presence of musculoskeletal symptoms had a significant negative correlation with the domains PF, RP, BP, SF and RE. This suggests that the physical and mental components of health-related quality of life were affected by musculoskeletal symptoms.
